# From Erythroblasts to Mature Red Blood Cells: Organelle Clearance in Mammals

**DOI:** 10.3389/fphys.2017.01076

**Published:** 2017-12-19

**Authors:** Martina Moras, Sophie D. Lefevre, Mariano A. Ostuni

**Affiliations:** UMR-S1134 Integrated Biology of Red Blood Cell, INSERM, Université Paris Diderot, Sorbonne Paris Cité, Institut National de la Transfusion Sanguine, Laboratoire d'Excellence GR-Ex, Paris, France

**Keywords:** erythropoiesis, mitophagy, organelle clearance, reticulocytes, enucleation, erythroblast maturation

## Abstract

Erythropoiesis occurs mostly in bone marrow and ends in blood stream. Mature red blood cells are generated from multipotent hematopoietic stem cells, through a complex maturation process involving several morphological changes to produce a highly functional specialized cells. In mammals, terminal steps involved expulsion of the nucleus from erythroblasts that leads to the formation of reticulocytes. In order to produce mature biconcave red blood cells, organelles and ribosomes are selectively eliminated from reticulocytes as well as the plasma membrane undergoes remodeling. The mechanisms involved in these last maturation steps are still under investigation. Enucleation involves dramatic chromatin condensation and establishment of the nuclear polarity, which is driven by a rearrangement of actin cytoskeleton and the clathrin-dependent generation of vacuoles at the nuclear-cytoplasmic junction. This process is favored by interaction between the erythroblasts and macrophages at the erythroblastic island. Mitochondria are eliminated by mitophagy. This is a macroautophagy pathway consisting in the engulfment of mitochondria into a double-membrane structure called autophagosome before degradation. Several mice knock-out models were developed to identify mitophagy-involved proteins during erythropoiesis, but whole mechanisms are not completely determined. Less is known concerning the clearance of other organelles, such as smooth and rough ER, Golgi apparatus and ribosomes. Understanding the modulators of organelles clearance in erythropoiesis may elucidate the pathogenesis of different dyserythropoietic diseases such as myelodysplastic syndrome, leukemia and anemia.

## Introduction

Mature red blood cells (RBCs) result from a finely regulated process called erythropoiesis that produces 2 million RBCs every second in healthy human adults (Palis, 2014). The standard model of erythropoiesis starts with hematopoietic stem cells (HSCs) in the bone marrow (BM), giving rise to multipotent progenitors that go on to erythroid-committed precursors to mature RBC. This hierarchical relationship is, however, challenged, showing a greater plasticity for the cell's potential fates, with several studies in mice (Adolfsson et al., 2005) and recent new data in human (Notta et al., 2016).

Maturation from erythroid-committed precursors is called terminal erythropoiesis and occurs in the BM within erythroblastic islands, which consist of a central macrophage surrounded by erythroblasts, and ends in the blood stream where reticulocytes complete their maturation within 1–2 days. During this phase, proerythroblasts (Pro-E) undergo morphological changes, such as cell size reduction and chromatin condensation, produce specific proteins, such as hemoglobin, and exhibit a reduced proliferative capacity to give rise to basophilic (Baso-E), polychromatophilic (Poly-E) and orthochromatophilic (Ortho-E) erythroblasts, successively. Even though several growth factors are known to regulate erythropoiesis, Epo is the main regulator of erythropoiesis driving RBC precursor proliferation and differentiation, preventing erythroblast apoptosis (Koury and Bondurant, 1990; Ji et al., 2011). The macrophage-erythroblast interaction in the BM is essential since macrophages facilitate proliferation and differentiation and provide iron to the erythroblasts (de Back et al., 2014).

At the end of the terminal maturation, mammalian erythroblasts expel their nuclei and lose all their organelles, such as the Golgi apparatus, endoplasmic reticulum (ER), mitochondria and ribosomes. After expelling its nucleus, the reticulocyte maturation continues, losing 20–30% of the cell surface (Waugh et al., 1997; Da Costa et al., 2001) and eliminating any remaining membrane-bound cytosolic organelles through an autophagy/exosome-combined pathway (Blanc et al., 2005).

**Figure 1:**
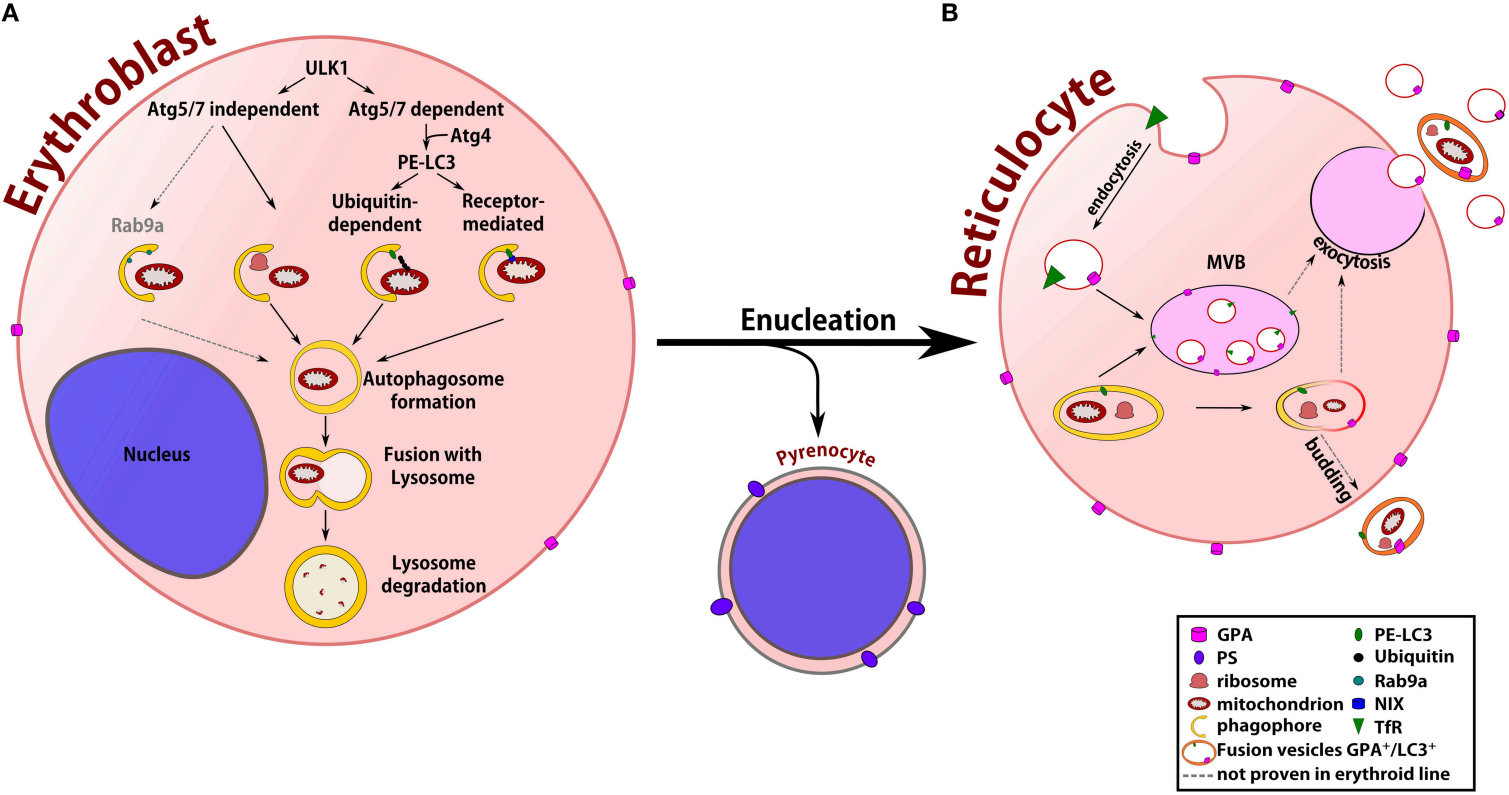
Terminal maturation of erythroblasts. **(A)** At the erythroblast stage, two Ulk1-mediated autophagic pathways are activated to allow organelle clearance: the Atg5/7-dependent pathway with the proteolytic Atg4-dependent activation of MAPLC3, microtubule-associated protein 1 light channel 3 (LC3) and the Atg5/7-independent pathway, which is not related to the LC3 protein. LC3 activation allows its insertion into the phagophore membrane, starting the engulfment of organelles through the recognition of an ubiquitin signal or by the direct binding of specialized receptors at the organelle membrane. In non-erythroid cells, Rab9a is important for the formation of the phagophore during the Atg5/7-independent autophagic pathway. After the formation of the autophagosome, its fusion with the lysosome permits the degradation of organelles by hydrolytic enzymes. The enucleation process gives rise to the pyrenocyte and the reticulocyte, which still contains some organelles that must be eliminated for the final maturation into erythrocyte. **(B)** During this stage, unwanted membrane proteins, such as transferrin receptor (TfR), are internalized by endocytosis and expelled by exocytosis from multi-vesicular body structures. Glycophorin A (GPA)/LC3 double-positive vesicles containing organelle remnants are also found in reticulocytes, suggesting cooperation between the endocytosis (GPA^+^) and autophagy (LC3^+^) pathways to eliminate organelles. How autophagosomes interact with multivesicular bodies (MVBs) following the same pathway of membrane protein recycling or budding directly from the plasma membrane after fusion with endocytic vesicles, however, remains unknown.

While extensive literature is done concerning the general mechanisms of erythropoiesis (Palis, 2014), this review focuses on the mechanisms and molecular actors involved during organelle clearance and membrane remodeling in order to produce fully functional biconcave mature RBCs. Figure 1 summarizes the best characterized steps of organelle clearance throughout erythroblast terminal differentiation.

## Enucleation

The most spectacular aspect of mammalian erythropoiesis is the generation of enucleated cells. Enucleation occurs in orthochromatic erythroblasts producing two kinds of cells, the reticulocyte and the pyrenocyte [the nucleus surrounded by a tiny layer of cytoplasm and the plasma membrane (PM)]. Pyrenocytes are rapidly eliminated by the macrophages of the erythroblastic island, where phosphatidylserine exposure acts as an “eat me” signal (Yoshida et al., 2005).

Among the changes occurring during terminal differentiation, cell cycle arrest, chromatin and nuclear condensation and nuclear polarization are important for enucleation. In addition, nucleus expulsion is believed to be dependent on adhesion protein reorganization across the PM and macrophage interactions (Lee et al., 2004; Soni et al., 2006). The transcription factor KFL1 is required for enucleation (Parkins et al., 1995; Magor et al., 2015), regulating the expression of cell cycle proteins, deacetylases, caspases, and nuclear membrane proteins (Gnanapragasam et al., 2016; Gnanapragasam and Bieker, 2017).

Nuclear and chromatin condensation is essential for enucleation (Popova et al., 2009; Ji et al., 2010) and is dependent on the acetylation status of histones H3 and H4 under the control of histone acetyl transferases (HATs) and histone deacetylases (HDACs). Accordingly, Gcn5, an HAT protein, is down-regulated, and H3K9 and H4K5 histone acetylation decreases during mouse fetal erythropoiesis. In addition, Gcn5 is up regulated by c-Myc, which is known to decrease during the late phase of the erythropoiesis (Jayapal et al., 2010). With the same model, the role of HDAC2 protein was shown to be essential not only for chromatin condensation but also for the formation of the contractile actin ring (CAR), which is involved in nuclear pyknosis (Ji et al., 2010). Moreover, it was recently shown that major histones are released through a nuclear opening that is induced by caspase 3 activity-dependent lamin B cleavage and chromatin condensation (Gnanapragasam and Bieker, 2017).

Many studies demonstrate the cell cycle dependence of enucleation (Gnanapragasam and Bieker, 2017). Interestingly, the cyclin-induced E2F-2 transcription factor, which is a direct target of KLF1 during terminal erythropoiesis, appears to play a role in enucleation by inducing the expression of CRIK (Citron Rho-interacting kinase). Away from its regular targets related to microtubule organization and cytokinesis, CRIK participates in nuclear condensation (Swartz et al., 2017).

Cytoskeletal elements play an important role in erythroblast enucleation, acting in a similar manner to cytokinesis but in an asymmetric way. Specifically, as observed by electron and immunofluorescence microscopy, actin filaments (F-actin) condensate behind the extruding nucleus to form the CAR. The use of cytochalasin D, an F-actin inhibitor, causes the complete blockage of enucleation (Koury et al., 1989). Furthermore, the formation of the CAR is dependent on Rac1 GTPase and on mDia2, a Rho GTPase downstream effector, since down-regulating these two proteins disrupts the CAR formation and blocks erythroblast enucleation (Ji et al., 2008).

Regarding other cytoskeleton elements, the pharmacological inhibition of vimentin does not affect enucleation, which is in agreement with its decrease during human erythropoiesis (Dellagi et al., 1983). However, the deregulation of microtubules diminishes the enucleation rate. Microtubules form a basket around the nucleus (Koury et al., 1989), which is displaced near the PM at the late erythroblast stages, suggesting that this network must be essential for the polarization of the nucleus. Recently, the importance of the molecular motor dynein, which mediates unidirectional movement toward the minus end of the microtubules, was shown. Furthermore, PI3K activity is induced by microtubule polymers, improves the polarization efficiency and promotes nuclear movement. However, PI3K inhibition does not block, but only delays, mice enucleation (Wang et al., 2012).

In 2010, Crispino's group observed, by electron microscopy, the formation of vesicles close to the nuclear extrusion site in both primary murine and human erythroblasts, suggesting that another mechanism contributes to enucleation. Additionally, as shown by genetic invalidation, clathrin is needed for the vesicle formation (Keerthivasan et al., 2010). More recently, it was shown that survivin is required for erythroblast enucleation, but instead of acting on cytokinesis via the chromosome passenger complex, survivin contributes to enucleation through an interaction with EPS15 and clathrin (Keerthivasan et al., 2012).

Clearly, we are still at the beginning of unraveling the molecular players involved in the enucleation process. Moreover, as shown in Table 1, most of the molecular players were identified in mice, and we are still lacking a demonstration that these players are also involved in human erythropoiesis.

**Table 1:** Comparison between studies in human or mice erythroid cells or in other cell models.

	**HUMAN erythroid cells**	**MICE erythroid cells**	**Other model or cell line**
**ENUCLEATION**			
PS exposition in pyrenocytes		Yoshida et al., 2005	
Role of KFL1	Magor et al., 2015	Parkins et al., 1995	
Role of Gcn5		Jayapal et al., 2010	
Formation of CAR		Ji et al., 2010	
Role of F-actin		Koury et al., 1989	
Role of Rac1 and mDia2		Ji et al., 2008	
Role of E2F-2		Swartz et al., 2017	
Role of dynein	Kobayashi et al., 2016		
Role of PI3K		Wang et al., 2012	
Vesicular trafficking	Keerthivasan et al., 2010	Keerthivasan et al., 2010	
Role of survivin/EPS15/clathrin	Keerthivasan et al., 2012	Keerthivasan et al., 2012	
Apoptotic involvement		Krauss, 2005	Weil et al., 1999
**MITOPHAGY**			
PINK1 accumulation			Narendra et al., 2010
Role of Parkin			Kim et al., 2008; Geisler et al., 2010
LC3 Cleavage	Betin et al., 2013		
Lc3B binding through p62			Pankiv et al., 2007
Engulfment inside the autophagosome		Koury, 2005	
Role of NIX	Aerbajinai et al., 2003	Zhang et al., 2012; Sandoval et al., 2008 Schweers et al., 2007	Yuan et al., 2017
Atg7 independent pathway		Honda et al., 2014; Mortensen et al., 2010; Zhang et al., 2009	
Role of FUNDC1			Chen et al., 2017
Role of Bcl2-L-13			Murakawa et al., 2015
Role of optineurin			Wong and Holzbaur, 2014
Role of prohibitin 2			Wei et al., 2017
KRAB/KAP1-miRNA regulatory cascade	Barde et al., 2013	Barde et al., 2013	
Role of 15-LOX			Kühn et al., 1990; Grüllich et al., 2001 Vijayvergiya et al., 2004
Role of Rab		Wang et al., 2016	Hammerling et al., 2017a,b
Role of hemin regulation	Fader et al., 2016		
Role of NF-E2	Gothwal et al., 2016	Gothwal et al., 2016	
**RIBOSOMES ELIMINATION**			
Role of Ulk1		Kundu et al., 2008	
Atg7-Independent pathway		Mortensen et al., 2010	
**PEROXISOME ELIMINATION**			
Role of macroautophagy			Iwata, 2006
Role of 15-LOX			Yokota et al., 2001
Role of Lon proteases			Yokota et al., 2008
**LYSOSOME ELIMINATION**			
Role of p62			Hung et al., 2013
**RNA ELIMINATION**			
Role of pyrimidin nucleotidase	Valentine et al., 1974; Lee et al., 2014		
**MEMBRANE REMODELING**			
TfR removing			Johnstone et al., 1989; Killisch et al., 1992
AQP removing			Blanc et al., 2009
α4β1 integrin removing			Rieu et al., 2000
GLUT and AChE removing			Johnstone et al., 1987
GPA^+^ exosomes	Griffiths et al., 2012		

### Mitochondrial clearance

The main mechanism for mitochondrial clearance is mitophagy, a selective type of autophagy that allows the degradation of damaged mitochondria. The importance of this process is highlighted by knowing that an impairment in mitochondrial function triggers an increase in reactive oxygen species production, which can in turn cause damage to cellular components (proteins, nucleic acid, and lipids) and trigger cell death (Lee et al., 2012).

During regular autophagy processes, stress or nutrient deprivation activates APM-activated protein kinase (AMPK), triggering two ubiquitin-dependent pathways (Figure 1A). One of these allows the assembly of the phagophore and involves several autophagy-related proteins (Atg), such as Atg5 and Atg7. The other aims to activate and lipidate LC3 (MAPLC3, microtubule-associated protein 1 light channel 3) by Atg4, a redox regulated protein. Atg4 and Atg7 cooperate to conjugate LC3 onto phosphatidylethanolamine in the lipid bilayer of the membrane originated from the ER-mitochondria contact site (Tooze and Yoshimori, 2010; Hamasaki et al., 2013). The elongated phagophore is then recruited to engulf targets via adaptor proteins, containing an LC3-interacting region (LIR) that forms a double-membrane autophagosome, which will fuse with a lysosome, initiating the degradation of the autophagosome components.

Upon mitochondria damage or depolarization, the mitochondrial membrane proteins are exposed and act as a beacon to recruit the phagophore membranes (Liu et al., 2014). An example is the PINK1 (P-TEN-induced kinase 1)-dependent recruitment of Parkin. Upon mitochondria depolarization, PINK1 accumulates at the OMM (outer mitochondrial membrane) and induces the mitochondrial translocation of Parkin, an RBR (ring-in-between)-type E3 ubiquitin ligase by direct phosphorylation (Kim et al., 2008; Narendra et al., 2010). The stabilization of Parkin at the OMM leads to the poly-ubiquitination of many proteins, inducing mitochondria fission and mobility stop and the phagophore recruitment by interacting with p62/SQSTM1, a LIR containing protein (Geisler et al., 2010). Unlike regular mitophagy induction, targeted mitochondria, during erythroblast maturation, are fully functional. BNIP3L/NIX, a BH3-only integral OMM protein first identified in mouse reticulocytes, appears to be the major mitochondrial protein involved during terminal differentiation (Schweers et al., 2007; Sandoval et al., 2008). This protein is upregulated during erythropoiesis and induces mitochondrial membrane depolarization and membrane conjugated LC3 recruitment to the mitochondria (Aerbajinai et al., 2003; Novak et al., 2010). Nix action is not mediated by its BH3 domain but rather seems to be due to a cytoplasmic short linear motif, acting as a cellular signal to recruit other proteins (Zhang et al., 2012). However, whether Nix-induced mitochondrial depolarization activates the Parkin-dependent pathway is still unknown (Yuan et al., 2017).

Recently, other mitochondrial receptors were found to participate in mitophagy, such as FUNDC1, induced by MARCH5, an E3 ubiquitin ligase acting in hypoxic condition (Chen et al., 2017), Bcl2-L-13 (Murakawa et al., 2015), optineurin (Wong and Holzbaur, 2014), and Prohibitin 2 (Wei et al., 2017). It remains unknown whether they play a role in erythroid maturation.

Canonical Atg proteins also participate in terminal maturation. In human erythropoiesis, LC3 cleavage is under the control of the endopeptidase Atg4 and is needed for autophagosome maturation (Betin et al., 2013). In mice, Ulk1 (Atg1) expression correlates with terminal differentiation and participates in mitochondria and ribosome elimination (Chan et al., 2007; Kundu et al., 2008). The ubiquitination-dependent pathway also plays a role in reticulocyte maturation but is not essential. Indeed, in Atg7^−/−^ reticulocytes, mitochondrial clearance is only partially affected (Zhang and Ney, 2009; Zhang et al., 2009). However, Nix and Ulk1 activation appears to be essential (Mortensen et al., 2010; Honda et al., 2014), suggesting the coexistence of both Atg5/Atg7-dependent and independent pathways during terminal differentiation.

Some studies suggest that the Atg5/7-independent degradation of mitochondria involves endosomal trafficking regulatory Rab proteins. Autophagosomes, formed in a Ulk1-dependent pathway, fuse with Golgi-derived vesicles and late endosomes in a Rab9a-dependent manner before they are targeted to the lysosomes (Wang et al., 2016). Interestingly, Rab proteins were also recently shown to be involved in mitochondria removal in a complete autophagy-independent pathway. Depolarized mitochondria appear to be engulfed in Rab5-positive endosomes that mature into Rab7-positive late endosomes and then fuse with lysosomes (Hammerling et al., 2017a,b). Unlike canonical autophagy, which involves the surrounding of a ubiquitin-decorated target by a double membrane structure, the entire mitochondria appears to be engulfed by an early endosome membrane invagination through the ESCRT machinery. Whether this might also occur in maturing erythroblasts is not known.

Mitophagy also appears to be transcriptionally regulated. Indeed, hemin-dependent differentiation of an erythroid cell line shows features of mitophagy (Fader et al., 2016). The NF-E2 transcription factor involved in globin gene expression also regulates mitophagy through the regulation of Nix and Ulk1 genes (Gothwal et al., 2016; Lupo et al., 2016). Another key regulator is the KRAB/KAP1-miRNA regulatory cascade, which acts as an indirect repressor of mitophagy genes in mice as well as in human cells, probably by the down and up regulation of a series of miRNAs, such as miR-351 that targets Nix (Barde et al., 2013).

In parallel to the autophagic pathway, cytosolic degradation seems to occur during reticulocyte maturation. 15-lipoxygenase (15-LOX), an enzyme that catalyzes the dioxygenation of polyunsaturated fatty acids, is translationally inhibited until the reticulocyte stage and acts to permeabilize organelle membranes, allowing proteasome access and degradation. Interestingly, only mitochondria elimination is affected, while ribosome clearance remains efficient when using a lipoxygenase inhibitor (Grüllich et al., 2001). This mechanism is still controversial, as 15-LOX might also act in the autophagy pathway as an OMM pH gradient disruptor that can induce mitophagy (Vijayvergiya et al., 2004), and on the oxidation of phospholipids conjugating with LC3 during the autophagosome formation; even so, these features, as shown in Table 1 were not demonstrated in erythroid cells yet (Morgan et al., 2015).

### Ribosomes and other organelles

In general, autophagy plays an essential role in the elimination of other organelles, such as lysosomes, peroxisomes and ER. However, the literature presents only very few studies in erythroid cells (Table 1).

While Nix is required for mitochondria removal, Ulk1 is involved in ribosome and mitochondria degradation (Schweers et al., 2007; Kundu et al., 2008; Sandoval et al., 2008). Similarly, an efficient clearance of ribosomes and ER and the inhibition of mitophagy was observed in Atg7^−/−^ mice (Mortensen et al., 2010). These data suggest that non-autophagic or Atg7-independent autophagic pathways might exist for the elimination of other organelles (Figure 1A).

In non-erythroid cells from mammals, it was proposed that peroxisomes are eliminated by three different pathways: macroautophagy (Iwata, 2006), 15-LOX mediated (Yokota et al., 2001) and the peroxisomal Lon proteases (Yokota et al., 2008). Furthermore, the autophagic degradation of lysosomes (lysophagy) was recently identified in HeLa cells where it is mediated by ubiquitination and involves p62 protein (Hung et al., 2013). The similarities between pexophagy/lysophagy and mitophagy in non-erythroid cells suggest that autophagy pathways might also be involved in erythroblast terminal maturation.

After enucleation, reticulocytes mature in the bone marrow (R1) and then exit in the blood stream (R2) to complete the process. While the degradation of organelles starts at the time of enucleation, the elimination of mRNA occurs in the blood stream and is mediated by ribonucleases, generating nucleotides that are degraded by the erythroid pyrimidine nucleotidase. This elimination is crucial, as the deficiency in this enzyme causes hemolytic anemia (Valentine et al., 1974). mRNAs in R2 reticulocytes mainly belong to three overlapping categories: transport, metabolic and signal transduction (Lee et al., 2014), and their presence is essential to reach the mature RBC stage. This supports the importance of the exosome pathway for the final maturation into RBCs with an active elimination of other subcellular components.

### Exocytosis and membrane remodeling

Exosomes are small vesicles that are secreted into the extracellular medium from various kind of cells. PM invaginations form early endosomes that engulf various targets forming multivesicular bodies (MVB, late endosomes) that eventually fuse with the PM and release exosomes. In reticulocytes, this pathway is thought to be involved in cell volume and membrane remodeling to reduce volume and remove unwanted membrane proteins. This was first discovered in sheep reticulocytes where transferrin receptor (TfR) is first internalized into small vesicles of 100–200 nm before being engulfed into the MVBs (Pan et al., 1985; Johnstone et al., 1989). The internalization step is clathrin-dependent, and the degradation is lysosome-independent and occurs by exocytosis after the fusion of the MVBs with the PM as shown in Figure 1B (Killisch et al., 1992). This process is required for the final elimination of other membrane proteins that are essential for the reticulocyte but are absent in the mature cell. Proteins such as aquaporin-1 (AQP1) (Blanc et al., 2009), α4β1 integrin (Rieu et al., 2000), glucose transporter and acetylcholinestarase (Johnstone et al., 1987) are found in glycophorine-A (GPA) positive endosomes while cytoskeletal proteins, such as actin or spectrin have never been found in these endosomes (Liu et al., 2010).

While plenty of evidence notes the role of autophagy in removing organelles during terminal maturation, the degradation step itself shows discrepancies with canonical proteolysis involving lysosomal proteins because of the disappearance of the lysosomal compartment during the maturation and removal of LAMP2 by exocytosis (Barres et al., 2010). Recently, GPA-positive endosomes were found to express LC3 at the endosome membrane, suggesting the cooperation of both autophagy and exocytosis in the removal of remnant organelles in R2 reticulocytes. These hybrid vesicles contain mitochondria, Golgi and lysosomes might be formed by the fusion of the outer-membrane of the autophagosome and the PM derived endosome (Griffiths et al., 2012). The exocytosis of this vesicle might be favored by the spleen, as splenectomized patients present large vacuoles inside reticulocytes (Holroyde and Gardner, 1970).

It should be pointed out the importance of lipids domain such as cholesterol and sphingomyelin-enriched domains in the PM remodeling, as they were find both in membrane vesiculation specific sites (Leonard et al., 2017).

## Conclusion

Even if all the animal models used to identify the molecular players involved during terminal differentiation exhibit maturation defects and anemia, links between organelle clearance and human hematological diseases are still mostly unknown. Erythroid disorders, such as β-thalassemia and myelodysplastic syndrome (MDS), are characterized by ineffective hematopoiesis, anemia, dissociation between proliferation and differentiation of progenitor cells and the inefficient elimination of aggregated protein (Arber et al., 2016; Taher et al., 2017). Indeed, defects in reticulocyte maturation and autophagy are identified in HbE/β-thalassemia patients (Lithanatudom et al., 2011; Khandros et al., 2012; Butthep et al., 2015), and enucleation defects are found in MDS patients (Garderet et al., 2010; Park et al., 2016). Impaired autophagy is involved in cytosolic toxic Lyn accumulation and mitochondria and lysosome degradation delay in chorea-acanthocytosis (Lupo et al., 2016). The use of autophagy modulators is beneficial in the case of SCD or β-thalassemia (Franco et al., 2014; Jagadeeswaran et al., 2017). Moreover, anemia in Pearson's syndrome was recently linked to incomplete mitochondrial clearance from reticulocytes (Palis, 2014) and an asynchronization of iron loading (Ahlqvist et al., 2015), while sickle cells patients showed an accumulation of proteins in their erythrocytes suggesting a defect in exosomal pathway (De Franceschi, 2009; Carayon et al., 2011).

Unraveling the molecular mechanisms and interplays ruling erythroblast terminal maturation would be priceless in hematological disease therapy. However, much of our knowledge regarding human erythropoiesis is based on animal models and/or *ex vivo* cultured human progenitor cells (Table 1). Great care should be applied when interpreting results, considering the important differences between mouse and human erythropoiesis as well as the *in vivo* and *in vitro* environments, as highlighted in the extensive transcriptome analysis across a terminal erythroid differentiation study (An et al., 2014).

## Author contributions

All authors listed have made a substantial, direct and intellectual contribution to the work, and approved it for publication.

### Conflict of interest statement

The authors declare that the research was conducted in the absence of any commercial or financial relationships that could be construed as a potential conflict of interest.
